# Interim Restorative Approach for the Management of Congenitally Missing Permanent Mandibular Incisors: Presentation of Three Cases

**DOI:** 10.1155/2011/717936

**Published:** 2011-07-14

**Authors:** Prashanth Prakash, Jayadev M. Hallur, Rachana Narse Gowda

**Affiliations:** ^1^Department of Pediatric Dentistry, Manubhai Patel Dental College and Oral Research Institute, Vadodra, Gugrat 390011, India; ^2^Department of Oral and Maxillofacial Pathology, Bapujji Dental College, Davangere, Karnataka 577002, India; ^3^Department of Dental Practice, Dr. M. R. Ambedkar Dental College, Bangalore, Karnataka 560092, India

## Abstract

Congenital missing of mandibular permanent incisors with retained primary incisors may jeopardize the esthetic appearance and psychological development of children, especially during the years of transition into adolescence. The retained primary teeth are necessary for the maintenance and normal development of alveolar bone, which in turn is essential for future definitive rehabilitation. In such situations, an interim restoration may be provided before any definitive treatment is given to comfort the young patient during this transition period. Interim restorations may include resin-modified additions to the existing teeth as well as more sophisticated restorations such as resin-retained bridge and removable partial dentures. However, this restoration differs for different clinical situations based on various factors such as age and patient compliance, and also consideration has to be given for the growth changes of the child. The aim of this present paper is to discuss the esthetic management of three cases with bilateral agenesis of permanent mandibular incisors and retained primary incisors with composite interim restoration.

## 1. Introduction

The agenesis of permanent teeth can seriously affect children both physically and emotionally, especially during the years of transition into adolescence. According to Hobkirk et al. 1994, the most common complaint of patients with hypodontia relates to their appearance or function, and most of them are detected at the age of 6–12 years, as late eruption is noticed by the dentist or commented upon by the patient or parent [1]. Interim restorations may be provided for such patients before definitive care is given to ease the transition of the child into early adolescence. These may include resin-modified additions to the existing teeth as well as more sophisticated restorations [2].

Hypodontia is the congenital absence of less than six teeth whereas oligodontia refers to congenital lack of more than six teeth excluding third molars [3]. There may be bilaterally missing teeth or unilateral [4]. Hypodontia is associated with the frequency of other missing teeth, the size of the remaining teeth, and the rate of dental development [2]. It is also associated with Down syndrome, cleft lip/palate, ectodermal dysplasias, Ellis-van Creveld, and incontinentia pigmenti. In the absence of other systemic conditions, both genetic component and environmental factors have to be considered as the etiology for isolated cases of hypodontia [5]. Local factors result in acquired hypodontia, for example, early irradiation of tooth germs, hormonal and metabolic influences, trauma, osteomyelitis, and unintended removal of a tooth germ during the extraction of a primary tooth [4]. Due to the agenesis of the permanent successor, the infraoccluded tooth does not exfoliate within the normal time range, and the root resorption is very slow. The clinician is in dilemma whether to retain the infraoccluded primary tooth or to extract it. Some possible treatment options include extracting the primary teeth followed by orthodontic space closure, or placing a space maintainer until final prosthetic reconstruction may be favorable [6]. In other situations, however, retaining the primary tooth may be the preferred mode of treatment. In these cases, buildup of the crown structure to maintain function and esthetics may be required. The crown build-up may be achieved via stainless steel crowns, esthetic posterior crowns, or resin-based composite restorations [7].

The high occurrence of congenitally missing mandibular incisor is seen in certain ethnic groups like Japanese, Korean, and Chinese. In Swedish population, lower central incisors are more commonly missing than in other races [2]. However, in certain other population it is as low as 0.23% for central incisor and 0.08% for lateral incisors [8]. This shows that there is a significant variation in the prevalence of missing mandibular incisors among different ethnic groups. Due to the lack of adequate data in this field, the multidisciplinary management of hypodontia is not well described. A retrospective study done by Davis in Hong Kong showed a high prevalence (3.4%) of missing mandibular incisors in this population [9]. Another study in Icelandic children showed a prevalence of missing mandibular central incisors and lateral incisors to be around 0.6% and 0.5%, respectively [10]. The difference in prevalence of congenitally missing mandibular incisors in different ethnic groups confirms the fact that both genetic and environmental factors are mainly involved [5].

The definitive replacement of missing teeth should be delayed until the eruption of adjacent permanent teeth and/or any necessary tooth movement has been completed. In the interim, the esthetic and functional demands of the patient can be met by the provision of composite additions, resin-retained bridge, or removable partial dentures. A removable partial denture significantly improves the appearance and function in patients with oligodontia. However, the provision of interim removable partial dentures should be avoided if at all possible and should only be considered in response to a specific complaint by the patient or when there is a clinical need. Their use in response to concerns and demands by the parents can often lead to failure through a lack of compliance by the child during treatment and space loss due to subsequent nonuse of the denture [11]. The definitive treatment of choice in case of hypodontia is the implant-supported prosthesis, but other options include conventional or resin-retained bridge involving the adjacent permanent teeth. Irrespective of the preferred definitive treatment, the space requirements aesthetically and functionally are similar for implant or bridge work. Therefore, it is required to maintain space mesiodistally and vertically for any of the definitive treatment options under consideration. If the adequate space is not maintained during the early periods of mixed dentition, it may jeopardize or limit the choice of the final restoration [11].

The term “interim restorations” is used to describe a restoration that has been placed in a tooth after the previous restoration, cracks and/or caries have all been removed at the commencement of endodontic treatment (i.e., the “investigation” stage of treatment). This aforementioned term is appropriate because of the connotation that “temporary” suggests a shorter time than “interim” [12]. These interim restorations can be used in patients with hypodontia before the provision of final definitive care for the patient. The interim management for patients with hypodontia varies according to the severity which includes composite additions, resin, retained-bridge veneers, onlays, and partial dentures which contribute to an improvement in aesthetics and function of the patient [2]. The timing and patient selection for using these treatment options reflect the needs and limitations imposed by the growing individual [11]. In this paper, we present three cases with congenital agenesis of permanent incisors and retained primary incisors which were treated successfully with composite, interim restorations to maintain space for future definitive treatment options along with enhancement of esthetics and function of the young patients.

## 2. Case Reports

**Figure 1:**
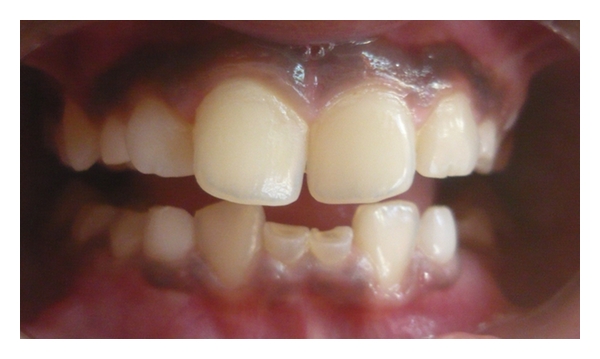
Frontal view showing retained and submerged primary incisors in an 11-year-old boy.

**Figure 2:**
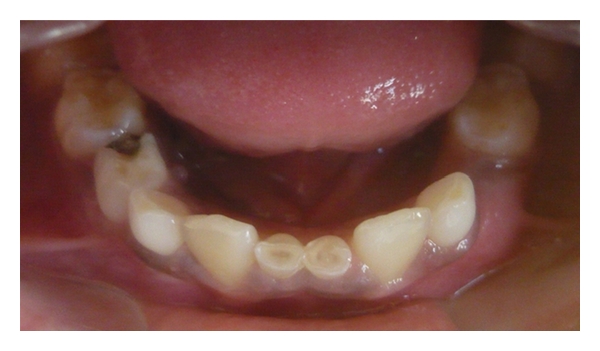
Retained primary incisors.

**Figure 3:**
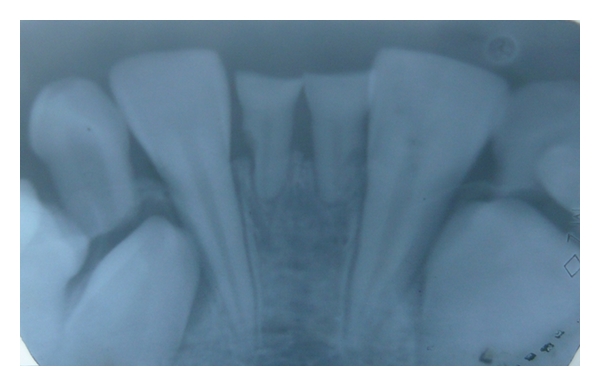
Radiograph shows congenitally missing permanent central incisors.

**Figure 4:**
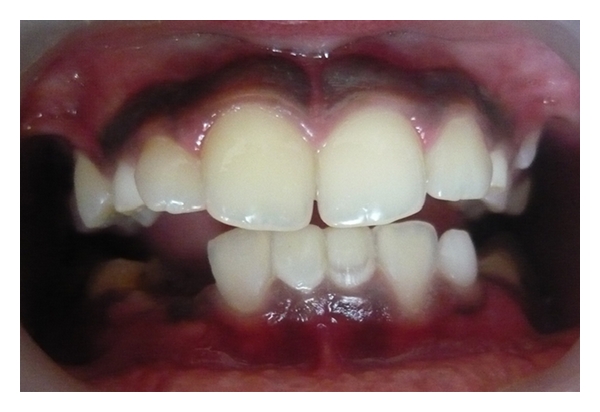
Composite interim restoration over retained primary incisors.

**Case 1 (see Figures 1, 2, 3, and 4)**

An 11-year-old male patient was referred to the division of pediatric dentistry with a chief complaint of retained milk teeth in the lower midline part of the oral cavity. The patient's mother was concerned about the unsightly appearance of the primary incisors. According to the mother, there was no history of trauma to the anterior region no history of infection or systemic conditions. There was no history of such findings in any of the patient's family members as well. The intraoral examination revealed the presence of retained primary mandibular incisors, and a radiograph in that region showed the absence of both permanent mandibular central incisors tooth buds (Figures 1, 2, and 3). The retained lower primary incisors were infra-occluded. One of the most important findings was the absence of any mobility associated with the primary incisors, and the teeth were vital. Although the radiograph showed the presence of external root resorption on both the teeth, there was no sign of mobility present in relation to both the primary incisors clinically. The radiograph also indicated the resorption of the mandibular deciduous canine roots due to the eruption of permanent canines which showed Grade 2 mobility clinically. The permanent maxillary central incisors, lateral incisors, first premolars, and first molars were erupted, and mandibular permanent lateral incisors and first molars were clinically present. Initially, the treatment plan was to extract the retained primary incisors and fabricate a removable partial denture, but because of the young age of the patient and the expected lack of co-operation by the patient to use the denture, the treatment plan was changed. A restorative approach was suggested to the parents, and consent was taken to proceed with the treatment. The primary incisors were prepared, isolated with cotton rolls, and followed by etching with 37% phosphoric acid for 15 seconds and bonding agent (Single Bond Adhesive System, 3M ESPE Adper Dental Products, St. Paul, Minn, USA) applied to the tooth surface. The curing of the composite was done with halogen light. Before restoration, any sharp margins on the prepared tooth surface were rounded to ensure that there was enough surface area for bonding. Also, a central pit or retention groove was prepared to increase the retention of the composite restoration (Z-100, 3M ESPE Dental Products, St. Paul, Minn, USA). Occlusal adjustments were done to prevent any premature contacts, and excess material was removed. After finishing and polishing with diamond burs and Soflex discs (3M ESPE Dental Products, St. Paul, MN, USA), the parents accepted the color of the restoration as compared with the adjacent normal teeth (Figure 4). Although the patient was recalled for other treatment procedures, the patient revisited the clinic only after three months. The restoration was intact, and there was no sign of tooth mobility at this visit. The patient was instructed to return immediately if there was any indication that the restored primary incisors were mobile.

**Figure 5:**
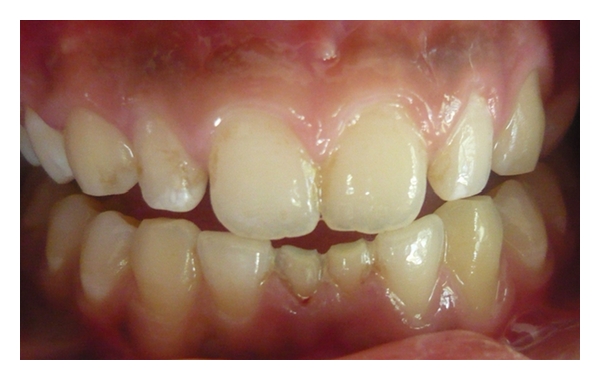
Submerged primary incisors in a 12-year-old child.

**Figure 6:**
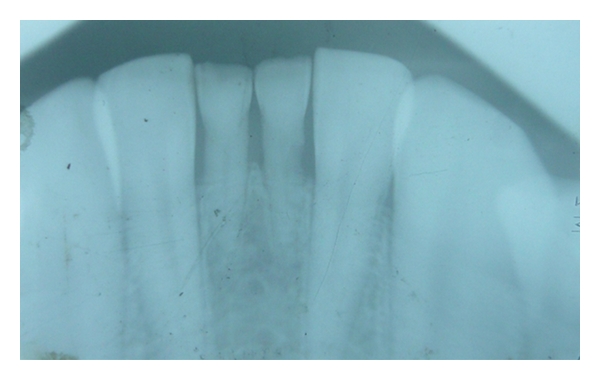
Radiograph shows congenitally missing permanent mandibular central incisors.

**Figure 7:**
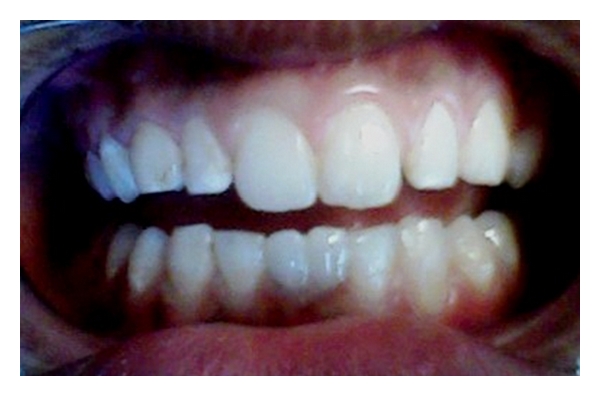
Esthetic composite interim restoration over retained primary incisors.

**Case 2 (see Figures 5, 6, and 7)**

A 12-year-old boy was reported with a complaint of discolored and retained deciduous teeth in the lower front teeth region. The patient was accompanied by his father, who gave a history of shedding of all other primary teeth except for the mandibular primary incisors, and was also concerned regarding the yellowish discoloration of the retained deciduous teeth (Figure 5). After intraoral examination, a radiograph was advised in the mandibular incisor region. Both clinical and radiographic examination confirmed the absence of permanent mandibular central incisors (Figure 6). In this case also, both the retained primary incisors were not mobile and were completely infra-occluded. The patient had Angle's Class I molar relation without crowding in the lower incisor region. The maxillary permanent incisors; canines; premolars; first molars were clinically present and permanent mandibular lateral incisors; canines; premolars; first molars were erupted except for the permanent mandibular central incisors. Periapical radiographs were taken in the mandibular posterior region to confirm that the central incisor was not migrated to posterior region of the arch. The patient's father was informed about the absence of both permanent central incisors and told about the possible treatment approach for such situations. The father of the patient was willing to go for a more esthetic approach and was not interested to get his child's intact teeth removed. Then, the treatment plan was to restore the retained primary incisors with composite, and the informed consent from the parents was taken to continue with our treatment. The submerged primary incisors were prepared by rounding off the sharp margins, and a central pit was prepared for better retention and bonding of the restorative material. The prepared tooth surface was etched with 37% phosphoric acid for 15 seconds and bonding agent (Single Bond Adhesive System, 3M ESPE Adper Dental Products, St. Paul, MN, USA) was applied followed by composite restoration (Z-100, 3M ESPE Dental Products, St. Paul, MN, USA) (Figure 7). Premature contact between the incisors was detected using articulating paper, and the excess was removed. The father of the patient appreciated the completion of treatment in a single visit rather than a multiple-visit procedures. As the patient did not come for follow-up visits, telephonic interview was done with the father of the patient after 3 months, which revealed that composite restoration was present without any fracture on the retained primary incisors. The father of the patient was advised to report to the clinic if there were any signs of mobility in the restored primary incisors.

**Figure 8:**
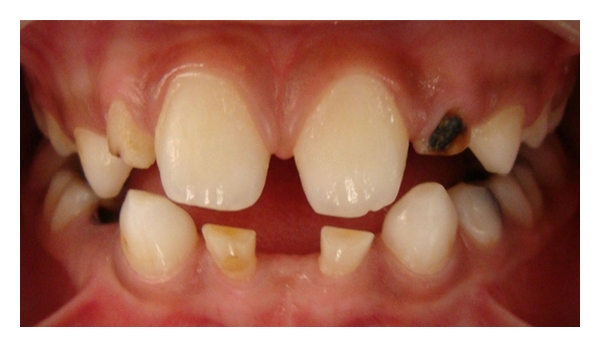
A 10-year-old boy with retained primary incisors.

**Figure 9:**
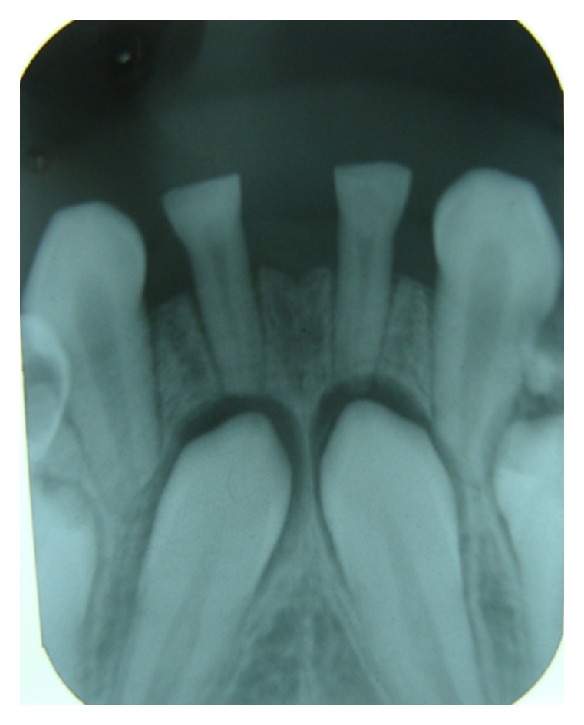
Periapical radiograph shows missing permanent mandibular incisors.

**Figure 10:**
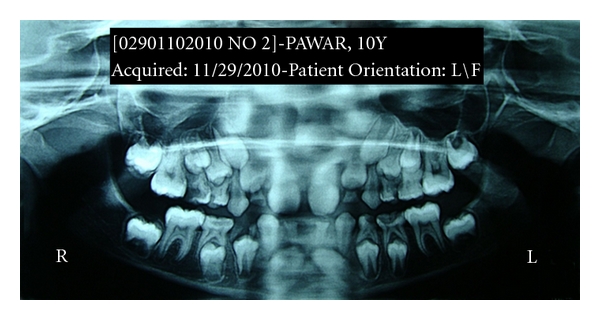
Panoramic view shows agenesis of permanent mandibular incisors.

**Figure 11:**
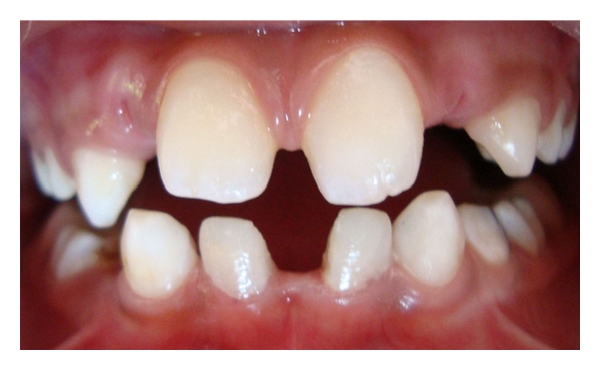
Composite interim restoration on retained primary incisors.

**Case 3 (see Figures 8, 9, 10, and 11])**

A 10-year-old boy reported tothe department of pediatric dentistry with a chief complaint of decayed teeth in the mandibular posterior teeth region, and the parent also showed concerns about the appearance of the mandibular anterior teeth. The patient did not have any significant medical or family history. The parents confirmed that none of the family members had congenitally missing permanent or deciduous teeth. On clinical examination, the patient had chronic irreversible pulpitis in relation to 55, 54, 64, 75, and 74. Dental caries was present in 65, 85, and 52, 62 showed grade 2 mobility. Root stumps were present in relation to 84 which was extracted and parents were advised to go for a fixed space maintainer. The important finding was the submerged primary mandibular lateral incisors present in the lower anterior region (Figure 8). A panoramic radiograph was advised which revealed the agenesis of permanent mandibular incisors (Figures 9, and 10). The parents were counseled about the missing permanent tooth buds, and the possible treatment options were suggested like extraction followed by removable space maintainer or composite interim restoration over the retained primary incisors. The parents and patient approved for the composite interim restoration which was more aesthetic and functional. The primary incisors were prepared with incisal rest seats on both teeth for better retention of the composite along with guide planes and positive finish lines. This was followed by etching with 37% phosphoric acid and adhesive application (Single Bond Adhesive System, 3M ESPE Adper Dental Products, St. Paul, MN, USA) over the prepared primary incisor. The composite (Z-100, 3M ESPE Dental Products, St. Paul, MN, USA) was applied in incremental layers, and every layer were light-cured separately. After the placement of adequate bulk of composite, reduction of excess material was done with diamond burs. The occlusal adjustments was done followed by finishing and polishing of the restoration with burs and Soflex discs (3M ESPE Dental Products, St. Paul, MN, USA) (Figure 11). The procedure was done in a single visit, and the patient was given appointment for future treatment. The patient never missed any future appointments, and all the required pulp therapy and restorations were done. The composite interim restoration was assessed during the 4-month follow-up visits which showed no signs of fracture or mobility of the teeth.

## 3. Discussion

According to Jones, there are three types of hypodontia which include mild (1 or 2 teeth missing), moderate (3–5 teeth missing), and severe (above 6 teeth missing) [13]. Hypodontia are also classified as isolated or nonsyndromic and syndromic or hypodontia associated with the syndromes [14]. The treatment option for mild to moderate forms of hypodontia includes space closure; space maintainance; space redistribution. 

The primary teeth act as ideal space maintainers that prevent the undesirable movement of adjacent teeth, which may cause difficulties in the placement of implants or fixed prosthesis [15]. The longevity of the retained primary teeth with no permanent successors is uncertain. A retrospective radiographic study done by Haselden et al. in 2001 has shown that some primary teeth without permanent successors can retain until fifties, but not beyond sixties. The recent trend of implant therapy suggests that the preservation of primary teeth until late teenage years is very critical. They have concluded that deciduous teeth should be retained where they can fulfill both esthetic and functional demands of the young patient as long as possible [16]. In the present paper follow-up examination conducted after 4 months showed no signs of resorption in the retained primary teeth. 

The ideal multidisciplinary team for management of hypodontia includes specialists from orthodontics, pediatric dentistry, prosthodontics, and implantology [2]. An early consideration of the likely definitive replacement for missing teeth forms the basis for the multidisciplinary management of hypodontia. The need to await complete eruption and root formation of permanent abutment teeth with huge pulp chambers has contraindicated the provision of fixed prosthetic reconstructions in children with hypodontia. The restorative team has an important role to play in the overall management of patients with hypodontia. The removable partial dentures and resin-retained bridgework has been used as interim restorations for missing teeth to maintain appearance and function [11]. The bulk of the prosthesis and potential movement of the removable partial denture is functionally and aesthetically unacceptable by young patients. This reflects both the reduced willingness of younger patients to accept dentures and the potential of removable prosthesis to harm the remaining permanent teeth over the long term and also hamper the lateral growth of the jaws in children [2, 11]. However, as esthetics was compromised with the use of dentures and abutment tooth preparation of adjacent permanent teeth was required for placement of resin-retained bridgework, we considered composite addition over the retained primary incisors would serve as interim restoration.

Improved techniques and materials have seen an increasing acceptance of composite restorations. Jepson and coauthors have suggested that anterior dental spacing can be improved using composite buildups and veneers [11]. The composite technique that restores the form and function with a minimal reduction of the tooth and conserves tooth material and good bonding of the resin-based composite with tooth structures is achieved. Therefore, composite restoration meets the functional and esthetic needs of the patient and is an easy, inexpensive solution for management of infra-occluded primary teeth. However, the patients must be warned about possible failure of the composite restoration, and the need for periodic follow-up appointments must be encouraged [7]. The composite strip crowns were not used because of the small size of the primary mandibular incisor crown forms which would not replicate the permanent mandibular incisors. Also, the height of the crown form would be short leading to infraocclusion of the restorations. The past reports of a particularly poor survival of interim restorations may be the result of the inadequacies of the then-available techniques and adhesive materials; another reason may be a more active lifestyle of such hypodontia patients. There is growing evidence of an improved survival rate of such restorations, when preparation of the tooth consisted of improved resistance form, thereby maximizing the bonding area. Such preparations include grooves, rest seats, guide planes, and positive finish lines [11]. 

The implant therapy with fixed prostheses is the well-accepted mode of definitive treatment for hypodontia, which fosters psychological well-being in such patients [2]. The implants have a high success rate and also avoid preparation of adjacent permanent teeth, in addition to maintaining appropriate interdental spaces [11, 13]. The fixture of implants in children is controversial, and many authors/clinicians consider it to be delayed until skeletal growth is completed [17]. The placement of implant-supported prosthesis is unlikely to be considered until clinical signs of growth cessation are present [11]. This provides maximum potential for implant placement without bone grafting and prevents infraocclusion of the ankylosed implant [2, 16]. Therefore, it is necessary for placement of interim restorations such as removable prosthesis; or resin-retained bridge work; or composite interim restorations until skeletal maturity is reached in children with missing permanent teeth [13]. 

## 4. Conclusion

The objectives of a hypodontia multidisciplinary team should be to provide improved esthetics, function, and occlusal stability. The development of different treatment options, which take into account growth and development of the dentition along with compliance of the child, can lead to a treatment plan that can produce acceptable interim results, which do not compromise any future definitive care. In conclusion, fixed composite interim restorations are preferable to removable ones to restore esthetics and function in young patients.
